# Bioinformatics and Experimental Validation for Identifying Biomarkers Associated with AMG510 (Sotorasib) Resistance in KRAS^G12C^-Mutated Lung Adenocarcinoma

**DOI:** 10.3390/ijms25031555

**Published:** 2024-01-26

**Authors:** Peng Lin, Wei Cheng, Xin Qi, Pinglu Zhang, Jianshe Xiong, Jing Li

**Affiliations:** 1 Key Laboratory of Marine Drugs, Laboratory for Marine Drugs and Bioproducts of Qingdao National Laboratory for Marine Science and Technology, Chinese Ministry of Education, School of Medicine and Pharmacy, Ocean University of China, Qingdao 266003, China; plin.r@foxmail.com (P.L.); wcheng.r@foxmail.com (W.C.);; 2 Faculty of Information Science and Engineering, Ocean University of China, Qingdao 266003, China

**Keywords:** lung adenocarcinoma, AMG510 (Sotorasib), resistance, single-cell transcriptomics, biomarkers

## Abstract

The Kirsten rat sarcoma viral oncogene homolog (KRAS)^G12C^ mutation is prevalent in lung adenocarcinoma (LUAD), driving tumor progression and indicating a poor prognosis. While the FDA-approved AMG510 (Sotorasib) initially demonstrated efficacy in treating KRAS^G12C^-mutated LUAD, resistance emerged within months. Data from AMG510 treatment-resistant LUAD (GSE204753) and single-cell datasets (GSE149655) were analyzed. Gene set variation analysis (GSVA) and gene set enrichment analysis (GSEA) were used to explore enriched signaling pathways, nomogram models were constructed, and transcription factors predicting resistance biomarkers were predicted. CIBERSORT identified immune cell subpopulations, and their association with resistance biomarkers was assessed through single-cell analysis. AMG510-resistant LUAD cells (H358-AR) were constructed, and proliferative changes were evaluated using a CCK-8 assay. Key molecules for AMG510 resistance, including *SLC2A1*, *TLE1*, *FAM83A*, *HMGA2*, *FBXO44*, and *MTRNR2L12*, were recognized. These molecules impacted multiple signaling pathways and the tumor microenvironment and were co-regulated by various transcription factors. Single-cell analysis revealed a dampening effect on immune cell function, with associations with programmed cell death ligand 1 (PDL1) expression, cytokine factors, and failure factors. The findings indicate that these newly identified biomarkers are linked to the abnormal expression of PDL1 and have the potential to induce resistance through immunosuppression. These results highlight the need for further research and therapeutic intervention to address this issue effectively.

## 1. Introduction

LUAD is one of the commonest malignancies [1,2,3]. Generally, conventional therapies used for LUAD include chemotherapy, radiotherapy, or a combination of both, and resistance to chemotherapy resulting in chemotherapy failure and death has become an ongoing serious medical problem [4,5]. *KRAS* stands as the predominant oncogene mutation found in humans, responsible for a quarter of all human cancers [6]. This oncogenic protein functions as a delicately regulated molecular switch, commanding a multitude of signaling cascades by binding either to its active (GTP) or inactive (GDP) state [7,8,9]. Mutations in this protein primarily arise from single amino acid substitutions at specific codons, commonly occurring at positions Q61, G12, or G13, with G12 mutations encompassing over 80% of all *KRAS* mutation cases [10]. The mutant-activated *KRAS* promotes tumor proliferation, metastasis, and invasion by stimulating multiple signaling routing pathways, such as the RAF-MEK-ERK signaling pathway [11], PI3K-AKT signaling pathway [12,13], and other signaling pathways [12,14].

Recently, the Food and Drug Administration (FDA) approved AMG510 [15], developed by Amgen, for the treatment of patients with advanced LUAD harboring G12C mutations in the *KRAS* gene [15]. AMG510 irreversibly binds to the substituted cysteine in KRAS^G12C^, locking *KRAS* in an inactive GDP-bound state and thus inhibiting downstream signaling [16]. The CodeBreaK100 Phase II single-arm trial revealed an objective response rate (ORR) of 37% and a median progression-free survival (PFS) of 6.7 months for sotorasib in advanced LUAD [17]. However, despite these achievements, resistance to AMG510 inevitably develops, and the mechanisms underlying this resistance are complex and remain largely unexplored. Therefore, we suggest that patients with lung adenocarcinoma being treated with AMG510 who develop resistance may have distinct gene expressions showcasing significant biomarkers that contribute to resistance development and facilitate tumor progression.

Although the KRAS^G12C^ mutation in LUAD has been targeted with AMG510, the mechanisms underlying the development of resistance to this treatment remain unclear, and the associated biomarkers have yet to be reported. In this study, we employed bioinformatics to identify biomarkers associated with AMG510 resistance in LUAD. We integrated data from multiple datasets and single-cell transcriptomics to thoroughly investigate the roles of these biomarkers in tumor progression. Furthermore, we constructed AMG510-resistant LUAD cell lines and experimentally validated the expression of these biomarkers within these cell lines.

## 2. Results

### 2.1. Identification and Functional Enrichment Analysis of the DEGs in a PDX Model of AMG510 Resistance of LUAD

**Figure 1:**
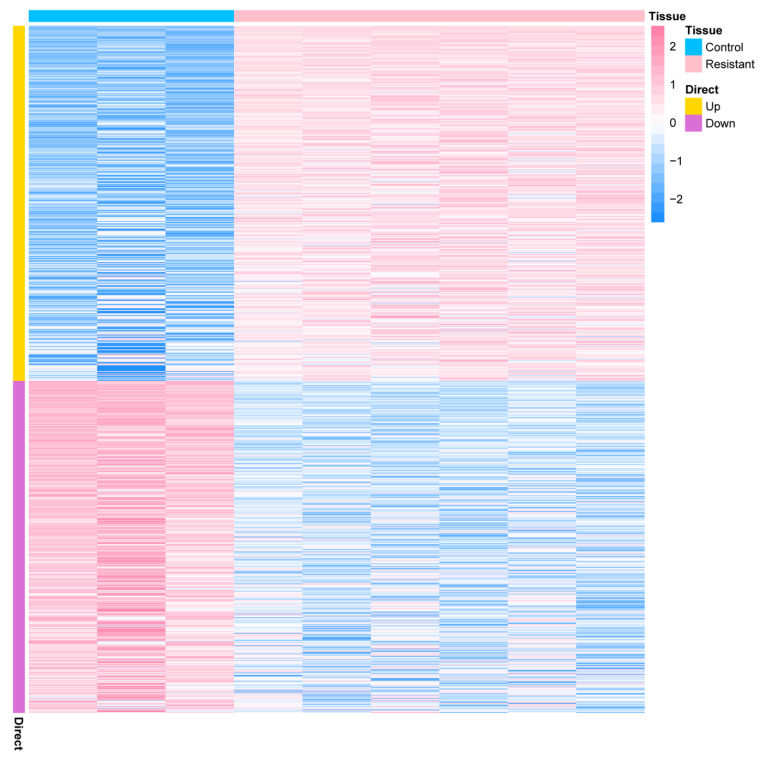
Heatmap visualization is presented, showing variations in gene expression between tissue samples from the control group (depicted in blue) and the drug-resistant group (demonstrated in pink) of LUAD patients. Elevated expression levels are represented in yellow, while a decline in expression levels is indicated by the color purple.

To investigate differentially expressed genes (DEGs) related to AMG510 resistance in LUAD, the GSE204753 dataset was accessed via the GEO database, which is open to the public. The criteria for considering DEGs included a low *p*-value (<0.05) and a significant absolute fold change (|logFC| > 1). Through this stringent selection, we identified 834 DEGs, consisting of 431 up-regulated and 403 down-regulated genes (Figure 1 and Table S2). Further exploration into the functional aspects of these 834 DEGs revealed connections with hormone responses, Golgi membrane functions, and adverse regulatory processes in the immune system (Figure S1).

### 2.2. Key Molecular Screening of AMG510 Resistance in LUAD

**Figure 2:**
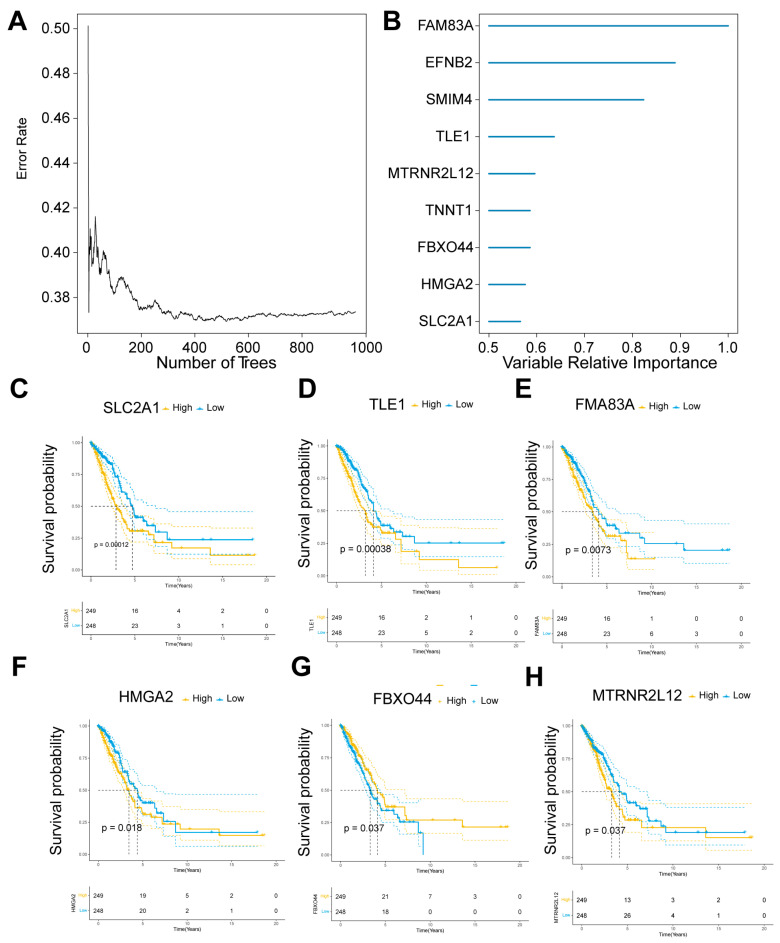
TCGA-LUAD cohort—DEGs were analyzed using a random survival forest approach. (**A**) This method was applied to scrutinize DEGs. (**B**) Within this set, nine genes stood out with variable relative importance markers exceeding 0.5. Kaplan–Meier survival plots revealed that (**C**) *SLC2A1* (*p* = 0.0012), (**D**) *TLE1* (*p* = 0.00038), (**E**) *FAM83A* (*p* = 0.0073), (**F**) *HMGA2* (*p* = 0.018), and (**H**) *MTRNR2L12* (*p* = 0.037) had high expression levels significantly linked to adverse overall survival outcomes. Conversely, a low expression level of (**G**) *FBXO44* (*p* = 0.037) was also determined to be significantly detrimental to overall survival.

To identify key molecules associated with resistance to AMG510 treatment in LUAD, we retrieved the mRNA expression data for LUAD patients from the TCGA database. After identifying 834 DEGs, we performed a random survival forest analysis and selected genes with a significance difference above 0.5 as key candidates. The analysis highlighted the importance of *FAM83A*, *EFNB2*, *SMIM4*, *TLE1*, *MTRNR2L12*, *TNNT1*, *FBXO44*, *HMGA2*, and *SLC2A1* (Figure 2A,B).

Next, we examined the association of these nine key candidate molecules with overall survival in LUAD. High expression levels of *SLC2A1*, *TLE1*, *FAM83A*, *HMGA2*, and *MTRNR2L12* were significantly correlated with reduced overall survival (*p* < 0.00012, *p* < 0.00038, *p* < 0.0073, *p* < 0.018, and *p* < 0.037, respectively). On the other hand, lower expression of FBXO44 was also significantly associated with decreased survival (*p* < 0.037) (Figure 2C–H). However, the expression levels of *EFNB2*, *SMIM4*, and *TNNT1* did not show a significant relationship with patient outcomes (Figure S2).

### 2.3. Key Molecules in AMG510 Resistance in LUAD Associated with Tumor Immune Cell Infiltration

**Figure 3:**
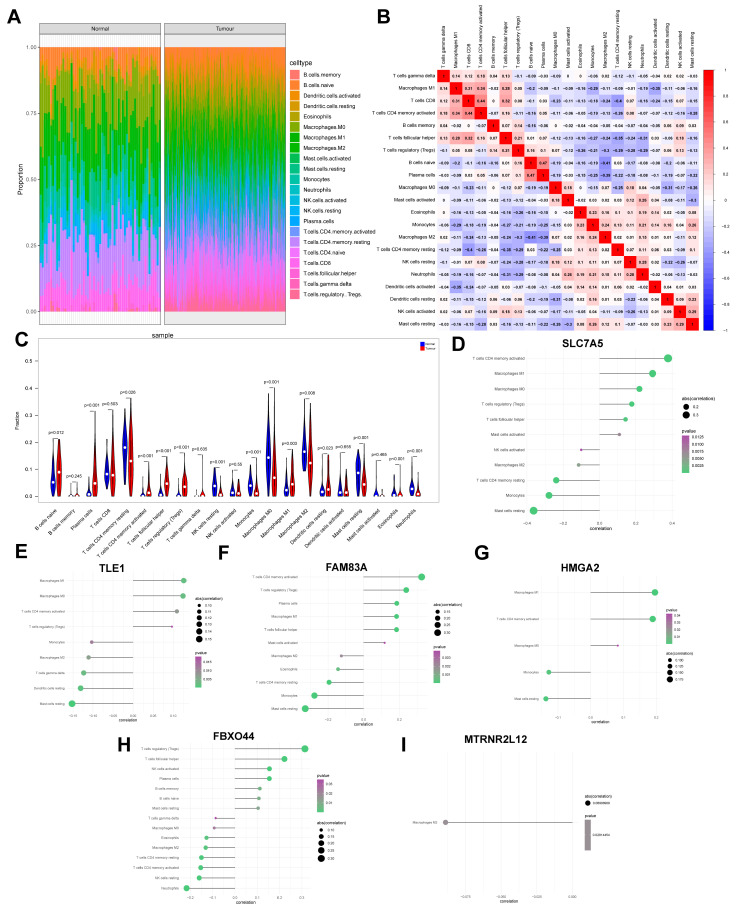
The composition of immune cells in normal control and LUAD group samples. (**A**) The immune cell makeup of the normal control group (shown on the left) was juxtaposed with that of the LUAD group (shown on the right). (**B**) During the pairwise comparison, we noticed significant correlations in the levels of immune cell infiltration between the normal control and the LUAD group. (**C**) A thorough analysis revealed variations in the expression levels of diverse immune cells across the normal control and LUAD groups. In addition, the study scrutinized the interplay between key drug resistance genes and immune cells. The genes under consideration were (**D**) *SLC2A1*, (**E**) *TLE1*, (**F**) *FAM83A*, (**G**) *HMGA2*, (**H**) *FBXO44*, and (**I**) *MTRNR2L12*.

To investigate the impact of key molecules associated with AMG510 resistance on immune infiltration in LUAD, we quantified immune cell populations in tumor and matched normal lung tissues of patients. The results showed significant differences in immune cell abundance between the tumor and normal groups (Figure 3A,B). Specifically, CD4 memory-activated T cells, naïve B cells, and plasma cells were found to be substantially elevated in tumor samples compared to normal samples (Figure 3C).

Furthermore, we conducted correlation analyses between immune infiltrating cells and key AMG510 resistance molecules. The findings revealed significant positive correlations between *SLC2A1* and CD4 memory-activated T cells, as well as M1 macrophages. Conversely, SLC2A1 exhibited significant negative correlations with resting mast cells and monocytes (Figure 3D). *TLE1* showed significant positive correlations with M1 macrophages and M0 macrophages, while displaying negative correlations with resting mast cells and resting dendritic cells (Figure 3E). *FAM83A* exhibited significant positive correlations with CD4 memory-activated T cells and regulatory T cells (Tregs), along with negative correlations with resting mast cells and monocytes (Figure 3F). *HMGA2* demonstrated significant positive correlations with M1 macrophages and CD4 memory-activated T cells, and negative correlations with resting mast cells and monocytes (Figure 3G). *FBXO44* showed significant positive correlations with Tregs and follicular helper T cells, while exhibiting negative correlations with neutrophils and resting NK cells (Figure 3H). Finally, *MTRNR2L12* displayed a significant negative correlation with M2 macrophages (Figure 3I).

### 2.4. Pathway Enrichment Analysis of Key Molecules for AMG510 Resistance in LUAD

**Figure 4:**
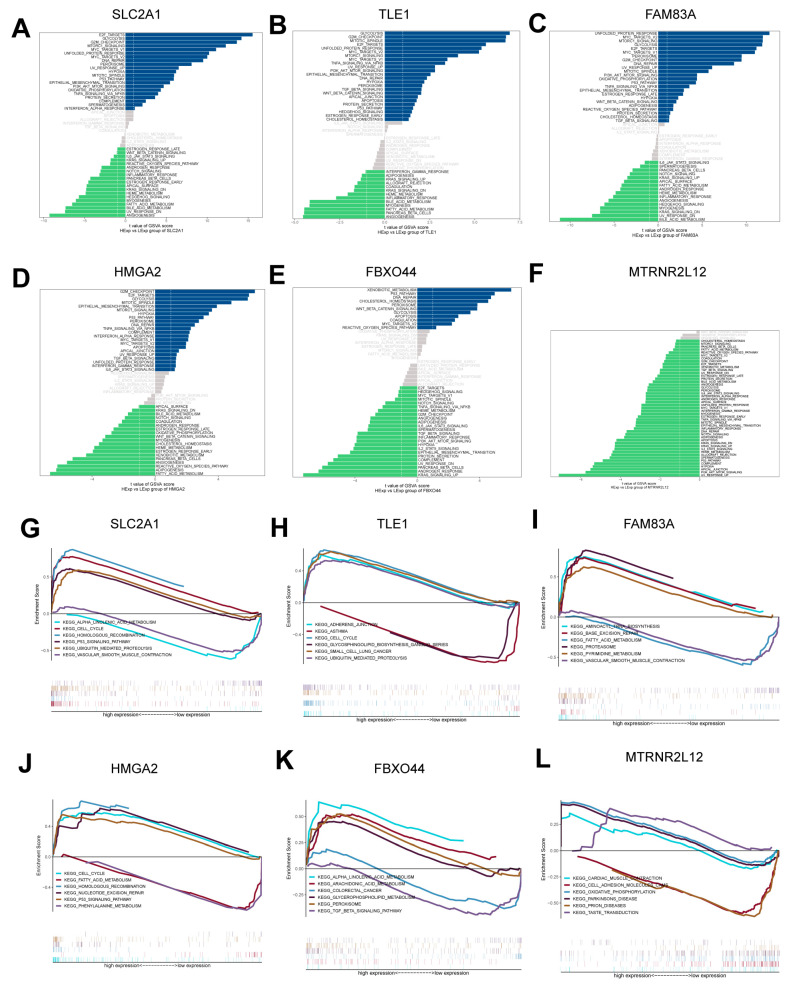
Key molecules of AMG510 resistance are associated with specific signaling pathways. (**A**) Signaling pathways enriched by *SLC2A1*, (**B**) *TLE1*, (**C**) *FAM83A*, (**D**) *HMGA2*, (**E**) *FBXO44*, and (**F**) *MTRNR2L12*. The results from gene set enrichment analysis (GSEA) indicate: (**G**) signaling pathways associated with *SLC2A1*, (**H**) *TLE1*, (**I**) *FAM83A*, and (**J**) pathways enriched through a high expression of *HMGA2*. Additionally, (**K**) *FBXO44* and (**L**) *MTRNR2L12* are associated with distinctive signaling pathways, as per GSEA results.

To understand the molecular mechanisms underlying the resistance to AMG510 treatment in LUAD, our study focused on the signaling pathways associated with key molecules. The GSVA results revealed significant associations of *SLC2A1* with pathways such as E2F targets, glycolysis, and the G2M checkpoint (Figure 4A and Figure S3). *TLE1* was predominantly linked to pathways like glycolysis, the G2M checkpoint, and mitotic spindle assembly (Figure 4B). *FAM83A* showed a strong association with pathways including myc targets, the unfolded protein response, and mTORC1 signaling (Figure 4C). *HMGA2* was notable for its involvement in pathways related to the G2M checkpoint, E2F targets, and glycolysis (Figure 4D). *FBXO44* was predominantly active in pathways such as xenobiotic metabolism, the P53 pathway, and DNA repair (Figure 4E). *MTRNR2L12* was linked to signaling pathways such as PI3K-AKT-mTOR signaling, UV response, and apical junction formation (Figure 4F).

Furthermore, GSEA data demonstrated that elevated *SLC2A1* expression primarily influenced cell cycle regulation, ubiquitin-mediated protein degradation, and P53 signaling pathways (Figure 4G). High levels of *TLE1* expression were associated with the reinforcement of cell-cycle-related signaling, the maintenance of adherens junctions, the ubiquitin–proteasome system, and pathways characteristic of LUAD (Figure 4H). Increased *FAM83A* expression was correlated with pathways governing aminoacyl-tRNA biosynthesis, base excision repair, proteasome activity, and pyrimidine metabolism (Figure 4I). Similarly, higher *HMGA2* expression was linked to the activation of the P53 signaling pathway, the enhancement of homologous recombination, the acceleration of cell cycle processes, and efficiency in nucleotide excision repair (Figure 4J). *FBXO44* expression specifically amplified pathways involved in arachidonic and α-linolenic acid metabolism, as well as glycerophospholipid metabolism and peroxisomal functions (Figure 4K). Lastly, a marked expression of *MTRNR2L12* was connected with improvements in cardiac muscle contraction, processes implicated in Parkinson’s disease, and mechanisms of taste transduction (Figure 4L).

### 2.5. Construction of Nomogram and Exploitation of Calibration Curves for Prediction of Prognosis of LUAD Patients by Key Molecules in AMG510 Resistance

**Figure 5:**
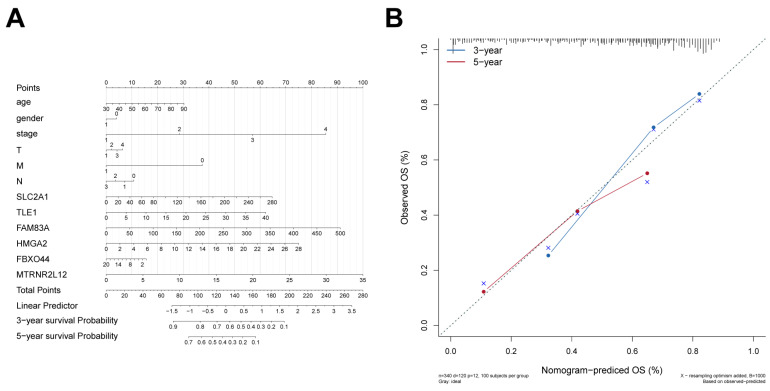
Nomogram and calibration curves for the prediction of the prognosis of LUAD patients. (**A**) The graph construction utilized the expression levels of *SLC2A1*, *TLE1*, *FAM83A*, *HMGA2*, *FBXO44*, and *MTRNR2L12* in conjunction with clinical parameters. (**B**) Calibration curves for the column line graph forecasts of 3-year and 5-year OS within the TCGA LUAD dataset.

To assess the value of AMG510-resistant pivotal molecules in LUAD prognosis, we developed a nomogram model to predict patient prognosis. In this model, logistic regression analyses demonstrated that clinical parameters (age, gender, and tumor stage) and core molecules (*SLC2A1*, *TLE1*, *FAM83A*, *HMGA2*, *FBXO44*, and *MTRNR2L12*) contributed differently to LUAD scores at different stages. Higher total scores predicted lower 3- and 5-year survival probabilities (Figure 5A and Figure S4). We then further developed calibration curves to validate the predictive accuracy of the nomogram model, and the results showed that it had a good predictive effect (Figure 5B).

### 2.6. Analysis of the Regulatory Network of Transcription Factors (TFs) Involved in Key Molecules of AMG510 Resistance

**Figure 6:**
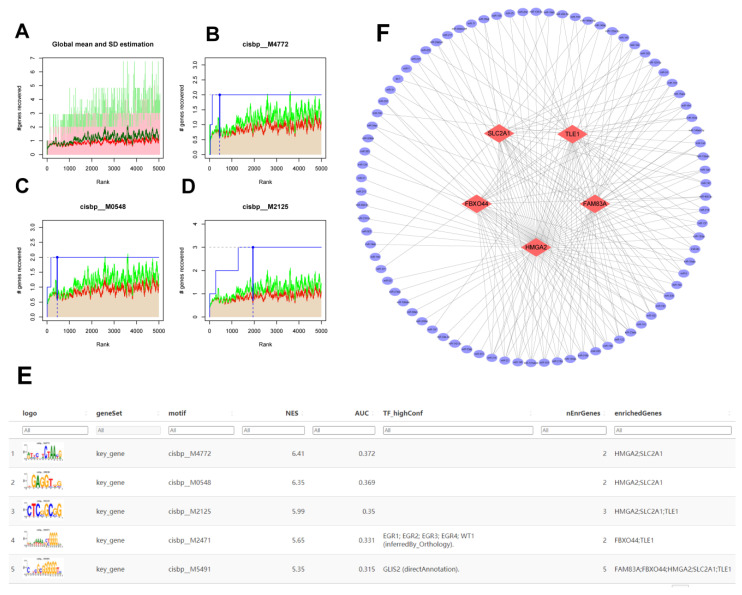
A Regulatory network analysis focused on key molecules of AMG510 resistance. (**A**) Enrichment analysis was performed to identify transcription factors associated with *SLC2A1*, *TLE1*, *FAM83A*, *HMGA2*, *FBXO44*, and *MTRNR2L12*. (**B**) We investigated the motifs attributed to cisbp_M4772, (**C**) the motifs linked to cisbp_M0548, and (**D**) the motifs related to cisbp_M2125. (**E**) We analyzed the enrichment of these motifs and their connection with resistance genes and their corresponding transcription factors. (**F**) A reverse prediction approach was used to identify 82 miRNAs and 201 mRNA–miRNA relationship pairs associated with biomarker resistance.

In this study, we examined the potential involvement of TFs in tumor progression by regulating specific molecules. Focusing on key molecules associated with AMG510 resistance, including *SLC2A1*, *TLE1*, *FAM83A*, *HMGA2*, *FBXO44*, and *MTRNR2L12*, we found they were regulated by multiple transcription factors. To delve deeper, we analyzed the enrichment of these TFs using cumulative recovery curves. The results revealed the motif with the highest normalized enrichment score (NES) was cisbp__M4772 (NES: 6.41) (Figure 6A–D), with *SLC2A1* and *HMGA2* being two of the genes enriched in this motif. Additionally, we analyzed the key molecules across all enriched motifs and identified the corresponding TFs (Figure 6E). Lastly, we used the mircode database for the reverse prediction of the key molecules, uncovering a total of 82 miRNAs and 201 mRNA–miRNA relationships, which we visualized using cytoscape (Figure 6F).

### 2.7. Correlation Analysis of Key Molecules in AMG510 Resistance with LUAD Oncogenes

**Figure 7:**
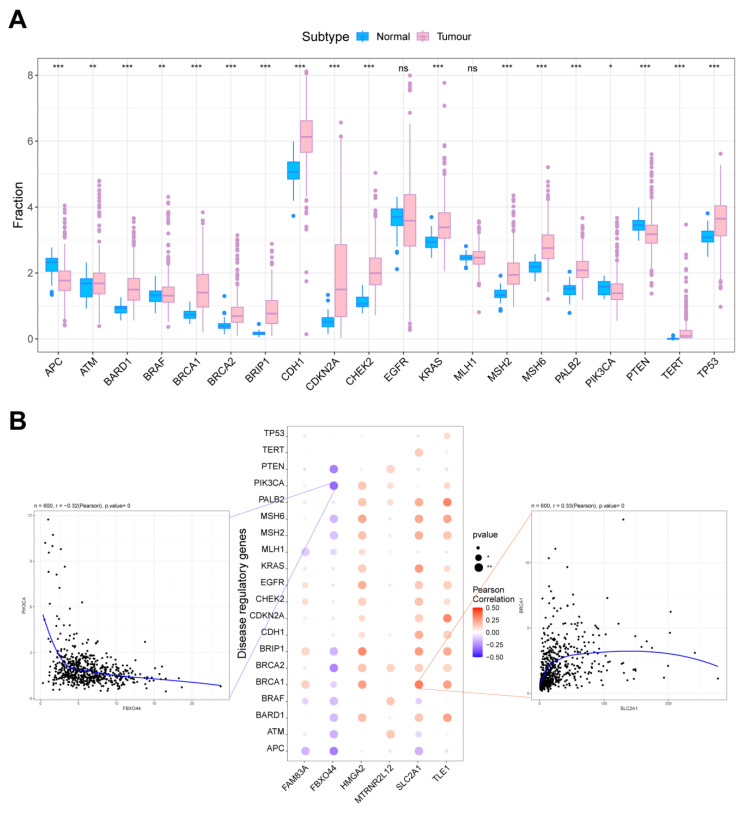
The relationship between key molecules in AMG510 resistance and LUAD-associated tumor genes. (**A**) The expression of tumor-related genes was compared between control subjects and LUAD patients. (**B**) Bubble plots illustrate the Pearson correlation between *SLC2A1*, *TLE1*, *FAM83A*, *HMGA2*, *FBXO44*, and *MTRNR2L12* and tumor-related genes, where the size of the circle indicates the proximity to a *p*-value of zero, a redder hue indicates a stronger positive correlation, and a darker purple signifies a stronger negative correlation. Asterisks denote significance levels (ns *p* > 0.05; * *p* < 0.05; ** *p* < 0.01; *** *p* < 0.001).

We obtained LUAD-related oncogenes through the GeneCards database (https://www.genecards.org/, accessed on 22 July 2023). The inter-group expression difference analyses for tumor genes implied that *APC*, *ATM*, *BARD1*, *BRAF*, and *BRCA1* gene expression varied among patients in these two groups (Figure 7A). These core molecules, as well as the top 20 mRNA gene expression levels, were analyzed as per the relevance scores. These critical molecule expression levels were observed to have obvious correlations with different tumor-associated genes’ expression levels, showing obviously positive correlations between *SLC2A1* and *BRCA1* (r = 0.33), as well as obviously negative correlations between *FBXO44* and *PIK3CA* (r = −0.32) (Figure 7B).

### 2.8. Single-Cell Analysis Revealed Correlations between Key Molecules in AMG510 Resistance and PD-L1, Cytokines, and Factors

**Figure 8:**
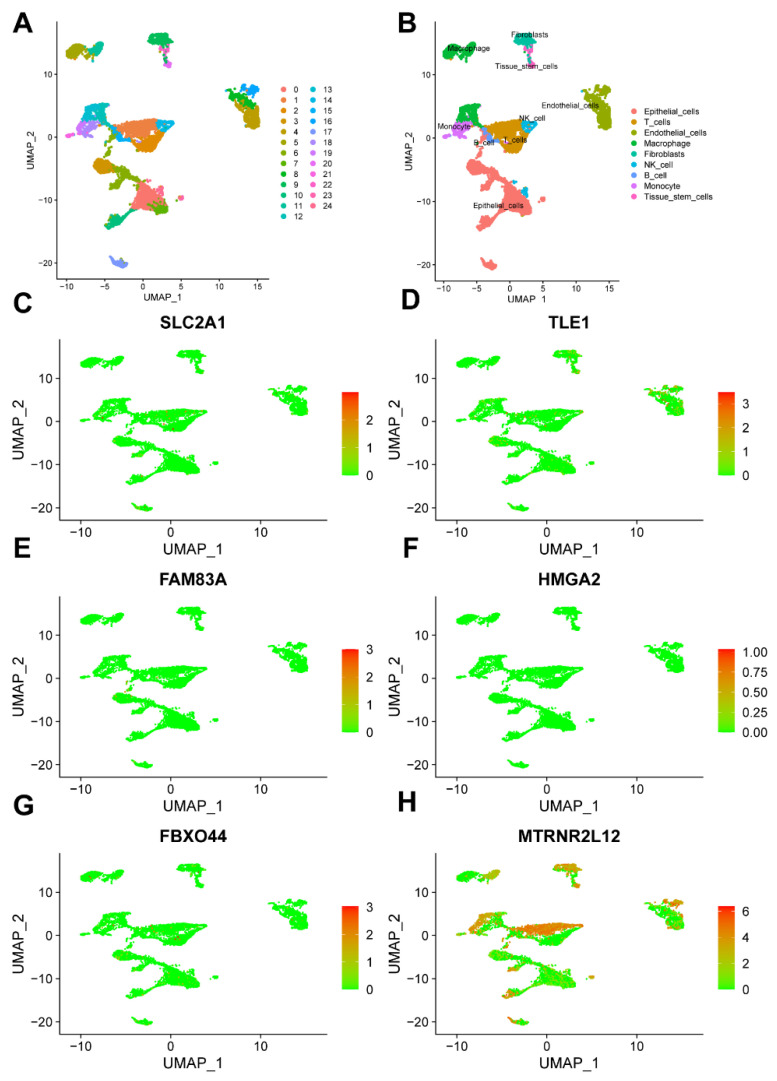
Single-cell analysis of key molecules in AMG510 resistance. (**A**) The UMAP algorithm was utilized for cell clustering, resulting in the identification of 25 distinct isoforms. (**B**) These 25 subtypes were categorized into the following cell types: epithelial cells, T cells, endothelial cells, macrophages, fibroblasts, NK cells, B cells, monocytes, and tissue stem cells. Key gene expressions in these nine cell types were examined: (**C**) *SLC2A1*, (**D**) *TLE1*, (**E**) *FAM83A*, (**F**) *HMGA2*, (**G**) *FBXO44*, and (**H**) *MTRNR2L12*.

Single-cell analysis unveiled the distribution of key molecules linked to AMG510 resistance in different cell types. Using the UMAP algorithm, we clustered single-cell data from lung adenocarcinoma into 25 subtypes (Figure 8A). Employing the SingleR package, we annotated these clusters into nine cell classes: endothelial cells, T cells, epithelial cells, NK cells, macrophages, fibroblasts, B cells, tissue stem cells, and monocytes (Figure 8B). The distribution of crucial genes within these classes was documented (Figure 8C–H and Figure S5).

**Figure 9:**
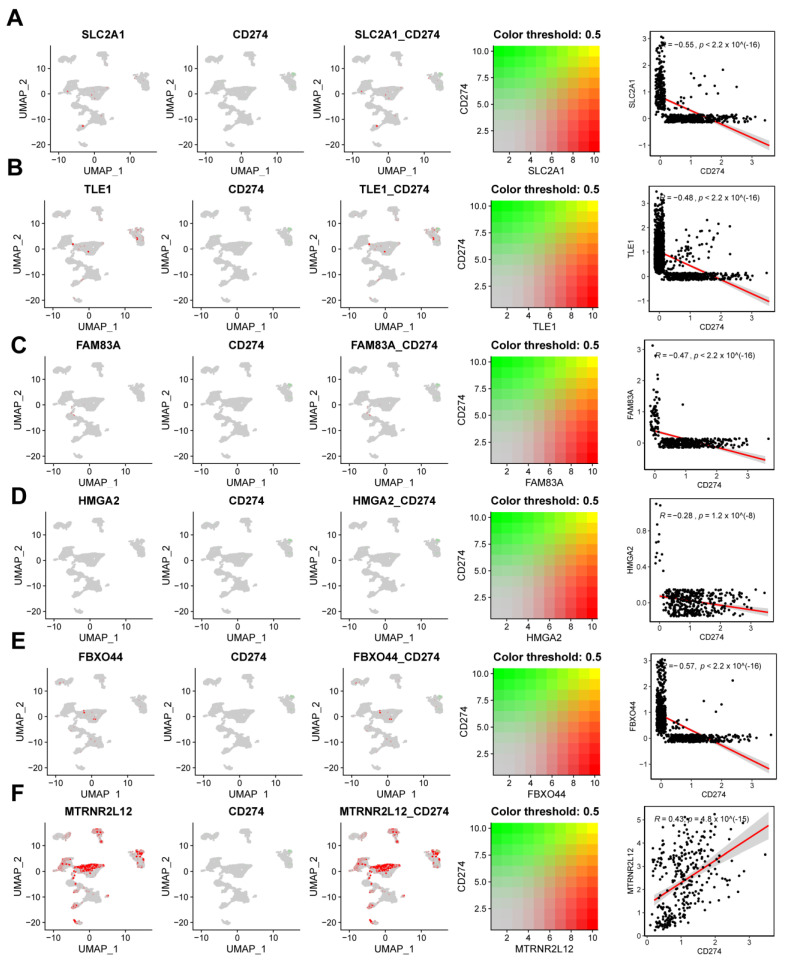
Single-cell analysis of the correlation between key molecules in AMG510 resistance and *CD274* (PDL1). (**A**) Expression of *SLC2A1* with *CD274* in the single-cell set of lung adenocarcinoma, with a correlation R of −0.55; (**B**) expression of TLE1 with *CD274* in the single-cell set of LUAD, with a correlation R of −0.48; (**C**) expression of *FAM83A* with *CD274* in the single-cell set of LUAD, with a correlation R of −0.47; (**D**) expression of *HMGA2* with *CD274* in LUAD single-cell sets with a correlation R of −0.28; (**E**) *FBXO44* with *CD274* in lung adenocarcinoma single-cell sets with a correlation R of −0.57; and (**F**) *MTRNR2L12* with *CD274* in LUAD single-cell sets with a correlation R of 0.43.

To investigate the correlation between AMG510 resistance molecules and *CD274* (PDL1) in tumor immune escape, we analyzed their co-expression patterns within these clusters. Our findings unveiled several significant correlations: *SLC2A1* showed a negative correlation with *CD274* (R = −0.55), as did *TLE1* (R = −0.48), *FAM83A* (R = −0.47), *HMGA2* (R = −0.56), and *FBXO44* (R = −0.57). Notably, *SLC2A1* showed a positive correlation with *CD274* (R = 0.43) (Figure 9A–F).

**Figure 10:**
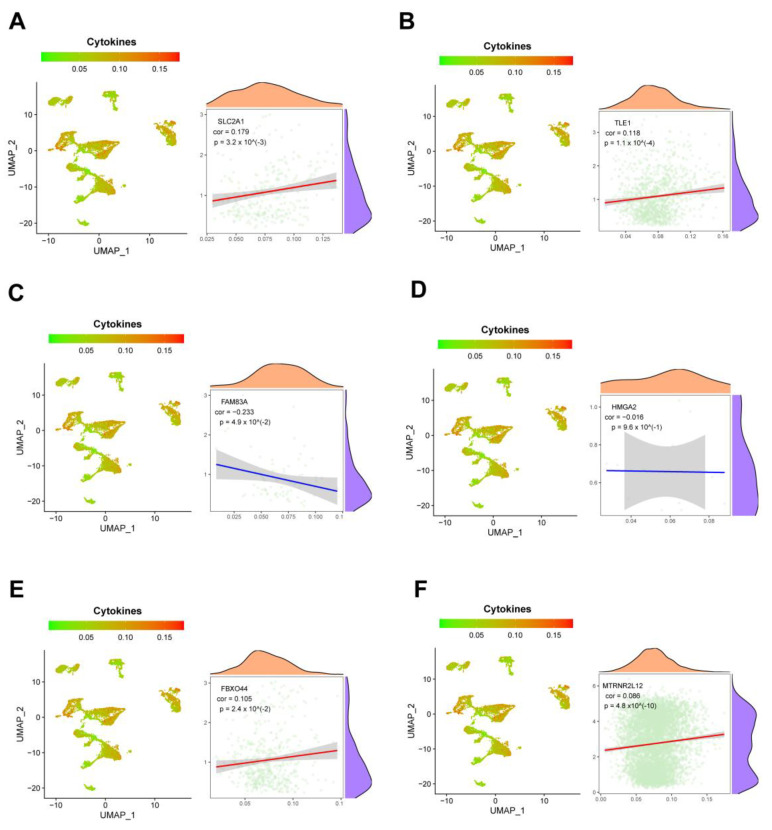
Single-cell analysis of the correlation between key molecules in AMG510 resistance and cytokine scores. (**A**) In LUAD single-cell set *SLC2A1* with a cytokine score R-value of 0.179; (**B**) single-cell set *TLE1* with a cytokine score R-value of 0.118; (**C**) single-cell set *FAM83A* with a cytokine score R-value of −0.223; (**D**) single-cell set *HMGA2* with a cytokine score R-value of −0.016; (**E**) the R-value of *FBXO44* versus cytokine fraction in the LUAD monoclonal cell set is 0.105; (**F**) the R-value of *MTRNR2L12* versus cytokine fraction in the LUAD monoclonal cell set is −0.066.

**Figure 11:**
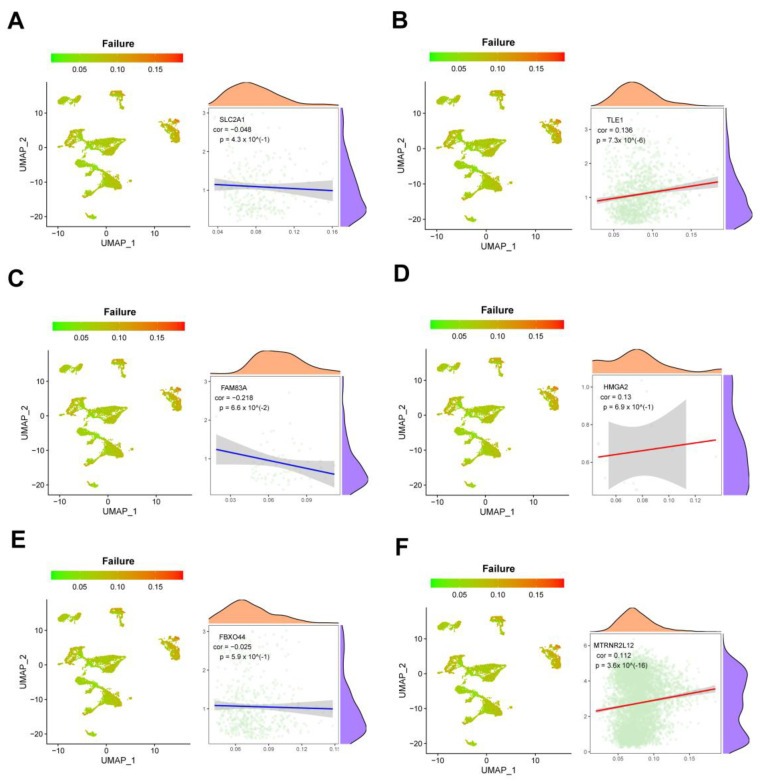
Single-cell analysis of the correlation between key molecules in AMG510 resistance and failure factor scores. (**A**) In the LUAD single-cell set *SLC2A1* with a failure factor score R-value of −0.048; (**B**) single-cell set *TLE1* with a failure factor score R-value of 0.138; (**C**) single-cell set *FAM83A* with a failure factor score R-value of −0.218; (**D**) single-cell set *HMGA2* with a failure factor score R-value of 0.13; (**E**) the R-value of *FBXO44* vs. failure factor score in the LUAD single-cell set is −0.025; (**F**) the R-value of *MTRNR2L12* vs. failure factor score in the LUAD single-cell set is 0.112.

At the single-cell level, we measured cytokine and exhaustion factor scores and analyzed their associations with important AMG510 resistance molecules. The analysis revealed significant positive correlations between cytokines and *SLC2A1*, *TLE1*, *FBXO44*, and *MTRNR2L12* (correlation values of 0.179, 0.118, 0.105, and 0.086, respectively) (Figure 10A,B,E,F). Conversely, *FAM83A* and *HMGA2* showed significant negative correlations with cytokines (correlation values of −0.233 and −0.016, respectively) (Figure 10C,D). In terms of the failure factor, *SLC2A1*, *FAM83A*, and *FBXO44* exhibited significant negative correlations (correlation values of −0.048, −0.216, and −0.025, respectively), while *TLE1*, *HMGA2*, and *MTRNR2L12* had significant positive correlations (correlation values of 0.136, 0.13, and 0.112, respectively) (Figure 11A–F).

### 2.9. Construction and Experimental Validation of the AMG510 Treatment-Resistant LUAD Cell Line

**Figure 12:**
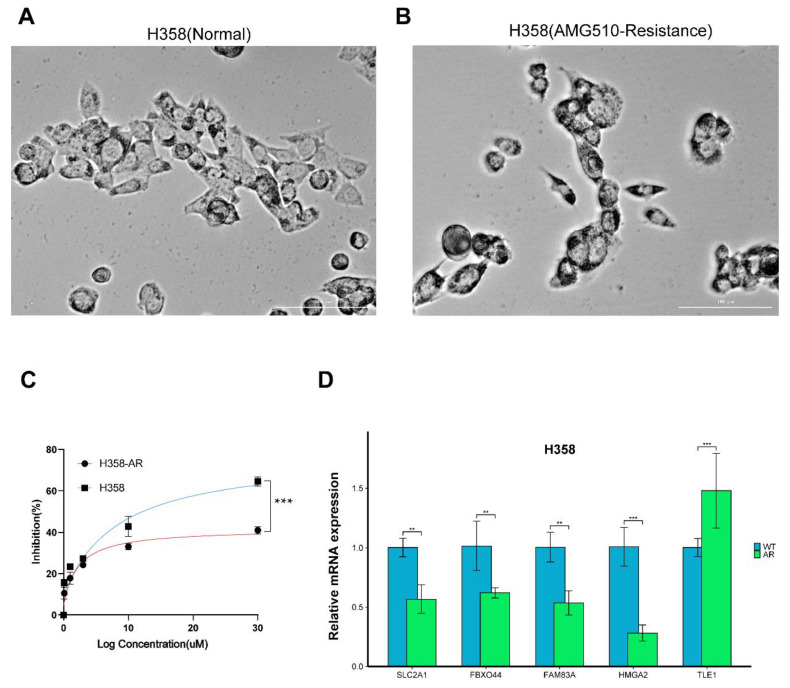
Construction and validation of resistant cell lines for LUAD bearing the AMG510 biomarker. (**A**) H358 and (**B**) H358-AR cell morphological differences. Scale bar: 100 μm. (**C**) Successful AMG510-resistant cell construction determined by comparing the IC50. (**D**) mRNA expression of drug resistance biomarker. *p*-value significant differences are indicated. (** *p* < 0.01; *** *p* < 0.001).

To explore AMG510 resistance biomarkers and their relationship with PDL1 in LUAD, we created an AMG510-resistant cell line (H358-R). Wild-type H358 cells exposed to prolonged AMG510 treatment transformed into a spindle shape (Figure 12A,B). A CCK-8 assay showed an enhanced tolerance in resistant cells (H358-R) compared to wild-type (H358) cells across different AMG510 concentrations (Figure 12C). qRT-PCR analysis revealed the downregulation of *SLC2A1*, *FAM83A*, *HMGA2*, and *FBXO44*, while *TLE1* was upregulated post resistance acquisition (Figure 12D).

**Figure 13:**
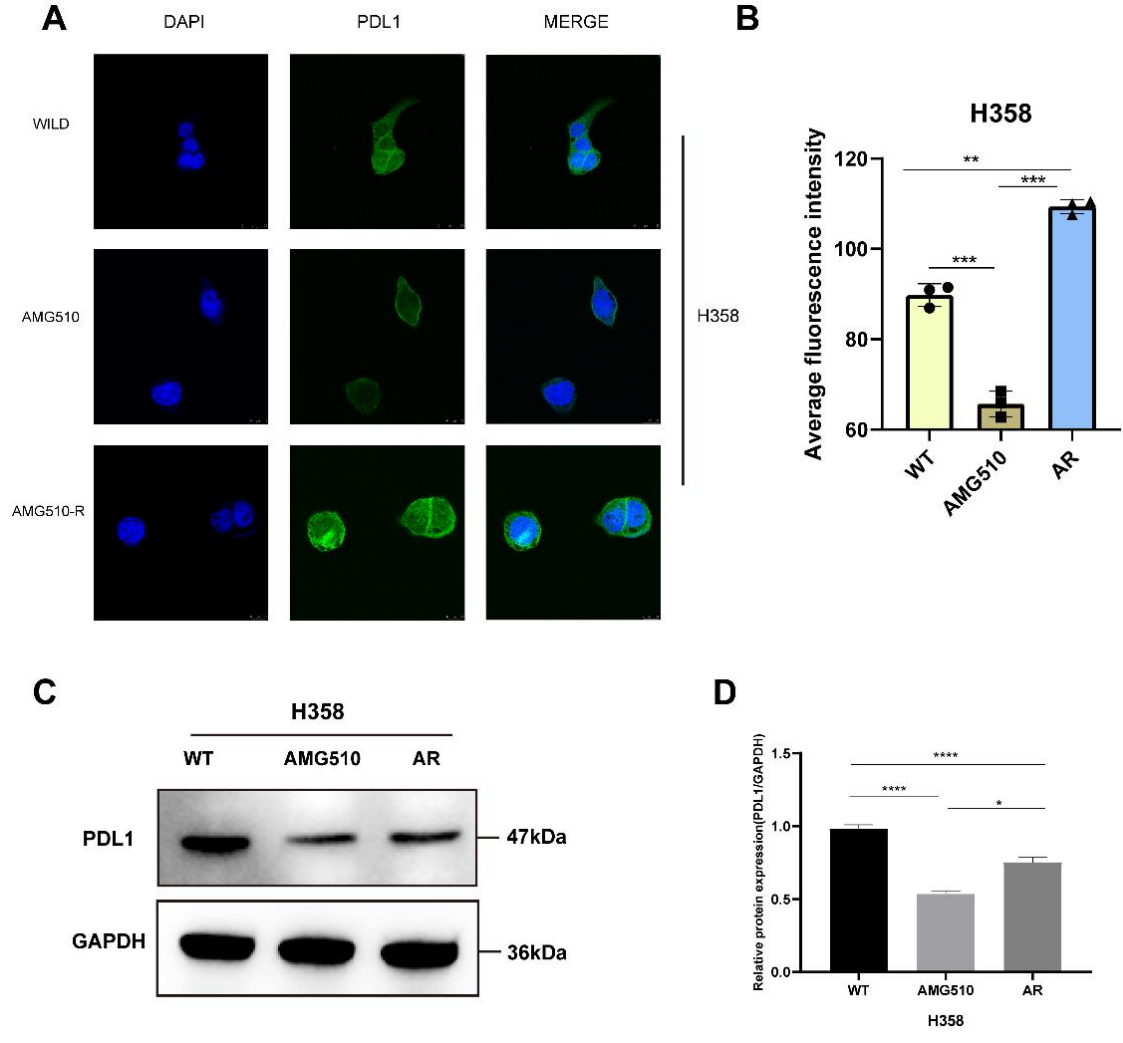
Experimental validation of key molecules in AMG510 resistance and PD-L1 expression in AMG510-resistant cells. (**A**) PD-L1 expression rose after AMG510 resistance. Cells were stained with anti-PD-L1 (green) and DAPI (blue). Scale bar: 10 μm. (**B**) Fluorescence intensity analysis of immunofluorescence. (**C**) PD-L1 protein expression was detected via WB in H358 cells and H358-AR cells treated with AMG510 or DMSO. (**D**) Grayscale analysis of PD-L1 relative protein expression in H358 and H358-AR cells (* *p* < 0.05; ** *p* < 0.01; *** *p* < 0.001; **** *p* < 0.0001).

The single-cell study indicated significant correlations between AMG510 resistance biomarkers and PDL1 in LUAD. Immunofluorescent staining showed decreased PDL1 expression in AMG510-treated H358 cells, while the resistant cell line had higher PDL1 expression than treated H358 cells (Figure 13A,B). Western blot analysis confirmed this pattern, with reduced PDL1 expression post AMG510 treatment and increased expression after acquiring resistance (Figure 13C); the changes were statistically significant (Figure 13D). In summary, we established the AMG510-resistant cell line (H358-R) and observed significant differences in key resistance biomarker expression between wild-type and resistant cells. PDL1 expression, linked to resistance biomarkers, also showed distinct differences between the two cell types.

## 3. Discussions

AMG510, a recently approved inhibitor for KRAS^G12C^-mutated LUAD treatment, offers improved outcomes for patients with the KRAS^G12C^ mutation. However, only a small portion of patients benefit from AMG510 due to inherent and acquired resistance, limiting its clinical utility [15,16,18]. Our study identifies biomarkers (*SLC2A1*, *TLE1*, *FAM83A*, *HMGA2*, *FBXO44*, and *MTRNR2L12*) associated with AMG510 resistance in KRAS^G12C^-mutated LUAD. These biomarkers exhibit significant expression changes related to AMG510 resistance and play crucial roles in tumor immunity, signaling pathways, and transcriptional regulation.

We found that multiple transcription factors may contribute to LUAD’s resistance to AMG510 treatment by jointly regulating the expression of genes such as *SLC2A1*, *TLE1*, *FAM83A*, *HMGA2*, *FBXO44*, and *MTRNR2L12*. This regulation reduces LUAD’s sensitivity and promotes resistance to AMG510 treatment. These crucial resistance biomarkers are primarily associated with signaling pathways like glycolysis and mTORC1. Previous research into cancer metabolism has established that abnormal cell proliferation in tumors relies on aerobic glycolysis, a trait shared by drug-resistant tumor cells. Furthermore, drug-resistant cells have elevated levels of byproducts of glycolysis, such as lactic acid, which aligns with the functions of our identified key biomarkers of drug resistance [19]. Another example is AT406, an antagonist of apoptotic proteins (IAPs), which effectively inhibits apoptosis in hepatocellular carcinoma cell lines. However, when mTOR is activated, it decreases the sensitivity of AT406 and leads to drug resistance in hepatocellular carcinoma [20,21]. The activation of mTOR signaling in drug-resistant cells is frequently reported [22,23], which is in line with the function of our essential resistance biomarker.

Transcription factors can drive resistance by modulating gene expression [24]. Among these, TLE refers to a member of the transcriptional co-repressor family, which includes TLE1-7 and is described as comprising the human homologs of Drosophila Groucho proteins [25]. It is of particular note that the level of TLE expression is critical for determining the sensitivity of tumor therapy. Shixiong Hu et al. found that the overexpression of *TLE2* increased the proportion of cells in the S-phase, and experimental verification showed that upregulated *TLE2* expression was correlated with increased sensitivity to gemcitabine [26,27]. In this study, *TLE1* was found to be highly expressed in AMG510-resistant PDX model mice. Furthermore, increased *TLE1* mRNA expression was detected in AMG510-resistant H358 LUAD cells. This is the first evidence demonstrating that TLE1-induced resistance to AMG510 occurs.

As single-cell technology platforms advance, we increasingly uncover the complexity of intra- and inter-tumor heterogeneity [28]. This heterogeneity arises from the reshaping of the tumor microenvironment, which induces changes in immune cell function and intercellular communication [29]. Ultimately, these alterations contribute to the development of drug resistance in tumors [30,31]. Although the prevailing view is that immune cells such as CD8^+^ T cells, CD4^+^ T cells, and NK cells perform anti-tumor functions in tumor therapy, due to tumor heterogeneity, these immune cells may be resistant to tumor therapy by regulating core immune checkpoint proteins and chemokines [32,33]. In the present study, we speculated that the biomarkers *SLC2A1*, *TLE1*, *FAM83A*, *HMGA2*, *FBXO44*, and *MTRNR2L12*, which are resistant to AMG510 treatment for LUAD due to tumor heterogeneity, significantly correlate with CD4 memory-activated T cells, M1 macrophages, Tregs, etc. Significant correlations affect the development of tumor therapy resistance, but the specific mechanisms need to be further demonstrated.

PD-L1 upregulation in tumor cells is a recognized mechanism for evading immune surveillance, contributing to tumor progression and therapy resistance [34]. Our single-cell analysis of lung adenocarcinoma found that the biomarkers of AMG510 resistance were present in immune cells and significantly correlated with PDL1 expression, suggesting an important role for these biomarkers in tumor progression and immune evasion. Moreover, these findings are reinforced by our study relating cytokine and exhaustion factor scores to the AMG510 resistance biomarkers, where specific biomarkers may serve as key immune response regulators or triggers of immune recovery. This study demonstrates a close correlation between the expression of key molecules associated with AMG510 resistance and PDL1, cytokines, and exhaustion factors within the monocytic populations of LUAD.

By establishing an AMG510-resistant LUAD cell line, we validated bioinformatic findings to confirm the differential expression of *SLC2A1*, *TLE1*, *FAM83A*, *HMGA2*, and *FBXO44* in wild-type and resistant cell lines. However, due to the pseudogene nature of *MTRNR2L12*, its expression was not detectable via RT-qPCR. While the work identified correlations between AMG510 resistance and PDL1, it did not delve into the biomarkers’ specific biological functions within LUAD nor the intrinsic mechanisms of regulation. Future investigations will aim to explore these aspects, potentially uncovering new insights into the coupling between these biomarkers and PDL1.

## 4. Materials and Methods

### 4.1. Data Downloads and Analyses

From the Gene Expression Omnibus (GEO) public database, we obtained a series matrix file (GSE204753) that is relevant to LUAD resistance. This file, named GPL21697, contains transcriptomic data for nine cases, which are divided into control (*n* = 3) and drug-resistant (*n* = 6) groups. To differentiate gene expression differences between these groups, we utilized the limma package with specific thresholds: *p*-values less than 0.05 and absolute log change (|logFC|) greater than 1. For the analysis of LUAD, we obtained both raw and processed mRNA expression data from the TCGA database (https://portal.gdc.cancer.gov/, accessed on 11 July 2023), which consisted of samples from the normal (*n* = 59) and tumor (*n* = 541) groups. Additionally, we acquired the single-cell dataset GSE149655 from the GEO public database, which includes four sets of single-cell data.

### 4.2. Randomized Survival Forest Analyses

The feature selection was conducted using the randomForestSRC package, and for ranking prognosis-related genes by importance, we applied the random survival forest algorithm, iterating it through a Monte Carlo simulation 1000 times (nrep = 1000). Furthermore, we identified genes exhibiting a relative importance exceeding 0.5 as final marker genes [35].

### 4.3. Immune Cell Infiltration Analyses

Patient information was processed using the CIBERSORT algorithm [36]. This tool was instrumental in estimating the relative proportions of 22 different types of immune cells infiltrating the tissue, and in executing Pearson correlation analyses linking gene expression and the contents of these immune cells [37].

### 4.4. GSVA Analyses

GSVA, a non-parametric and unsupervised method, is used to assess gene set enrichment in transcriptomic datasets [38]. By computing integrated scores for gene sets, GSVA transforms changes at the gene level into alterations at the pathway level, aiding in the identification of the biological functions of the samples.

### 4.5. GSEA Analyses

We conducted GSEA to categorize genes based on their differential expression between two sample types, utilizing a specific gene set [39]. This analysis assessed whether the predetermined gene set showed enrichment at either the top or bottom of our ranked list. In this study, by employing GSEA, we compared differences in Kyoto Encyclopedia of Genes and Genomes (KEGG) signaling pathways between groups with high and low expression to investigate the molecular mechanisms of biomarkers in patients from these groups.

### 4.6. Nomogram Modeling

A nomogram model is constructed based on regression analysis, incorporating clinical symptoms and the expression of key genes [40]. The predictive value is calculated by constructing a multifactorial regression model, assigning scores to each level of every influential factor according to their corresponding contribution to the outcome variable (size of the regression coefficient), and then summing the individual scores to obtain a total score.

### 4.7. miRNA Analyses

MicroRNAs (miRNAs) have a recognized role in controlling gene expression, whether through mRNA translation inhibition or mRNA degradation facilitation [41]. Our additional analysis sought to determine whether some miRNAs present within biomarkers could influence the degradation or transcription of specific genes associated with risk. Identification of miRNAs linked to biomarkers was accomplished by leveraging the miRcode database. Subsequently, we utilized Cytoscape (Version 3.8.2) software to visualize the networks between miRNAs and genes.

### 4.8. Regulatory Network Analyses of Biomarkers

The R package “RcisTarget” has been utilized to predict transcription factors [42]. Motifs serve as the foundation for all calculations executed using RcisTarget. In addition to motifs annotated by the source data, further annotations have been inferred based on gene sequences and motif similarity. The gene-motif ranking database “RcisTarget.hg19. motifdb.cisbp.500bp” has been utilized.

### 4.9. Single-Cell Analyses (SCAs)

The data were processed using the Seurat package, and the UMAP algorithm was applied to analyze the positional relationships between clusters [43]. The celldex package was used for annotations, with specific cells that have significant associations with tumorigenesis annotated accordingly. For each cell subtype, marker genes were extracted from single-cell expression profiles by setting the log-fold change threshold parameter (logfc.threshold) of the “FindAllMarkers” function to 1.

### 4.10. Cell Cultures and Reagents

H358-AR denotes an AMG510-resistant LUAD cell line, while H358 represents a specific type of LUAD cell line. H358 cell lines used in this study were obtained from our laboratory’s cell bank and have been previously utilized in this experiment. For culturing, we used RPMI 1640 medium enriched with 1% penicillin/streptomycin (Basic Medium) and 10% fetal bovine serum (supplied by Excell Bio), maintaining the cells at 37 °C in a 5% CO_2_ environment. AMG510 was prepared in DMSO. Both H358 and H358-AR cells were independently plated in six-well plates and subsequently photographed post attachment, using an enzyme marker for clear identification.

### 4.11. CCK-8 Assay

H358 and H358-AR cells were cultured in 96-well plates, with an initial density of 5000 cells per well, and left to incubate overnight. The subsequent day saw the treatment of both cell types with varying concentrations of AMG510 (0, 0.1, 1.0, 3.0, 10.0, 30.0 µM) for a span of 24 h. Following this, the cells were subjected to an additional 3 h incubation, after which we measured the absorbance at 460 nm using a CCK-8 assay kit from TargetMol (Shanghai, China, topscience, Catalog #C0005).

### 4.12. Quantitative Reverse Transcription PCR (qRT-PCR)

Following the guidelines of the PrimeScript RT Reagent Kit (Osaka, Japan, TaKaRa, 9109), RNA extraction was carried out utilizing Trizol, succeeded by the process of reverse transcription. The PCR reactions were performed with the SYBR Premix Ex Taq Kit (Japan, TaKaRa, 9109), adhering strictly to the instructions provided by the manufacturer. The sequences of all primers used can be found in Table S1.

### 4.13. Immunofluorescence

We initiated our experiment by seeding H358 and H358-AR cells on slides, maintaining a density of 3000 cells in 100 µL of growth medium. After a period of 24 h, we treated the H358 cells with AMG510 at a concentration of 1 µM, followed by an additional 24 h incubation. The H358-AR cells, conversely, received only AMG510 treatment. Post fixation and membrane permeabilization, the cells underwent overnight incubation at 4 °C with PD-L1 (Guangzhou, China, CST, D8T4X) Rabbit mAb (Alexa Fluor 488 Conjugate, CST #25048) at a 1:100 dilution in PBS. The following day, the cells were cleansed and stained with 4′,6-diamidino-2-phenylindole (DAPI) for 15 min to enable nuclear visualization. Imaging was performed using a Leica SP8 STED 3X confocal microscope (Berlin, Germany, Leica), employing a 63× oil immersion objective for detailed observation.

### 4.14. Western Blotting

We initiated the experiment by culturing H358 and H358-AR cells overnight in 6-well plates, maintaining a density of 10,000 cells per well. The H358 cells were treated with both DMSO and AMG510 for a span of 24 h, while the H358-AR cells received only AMG510 treatment. Utilizing RIPA Lysis Buffer (Shandong, China, SparkJade, EA0002), we extracted the total protein and subjected it to analysis following standard Western blotting protocols. The antibodies incorporated in this procedure were PD-L1 (1:1000, Centennial, CO, USA, NOVUSBIO, 80490), and GAPDH (1:10,000, Woburn, MA, USA, ABclonal, AC002).

### 4.15. Statistical Analyses

All statistical analyses were conducted utilizing the R language. We performed every statistical test as a two-sided test, defining statistical significance as a *p*-value below 0.05.

## 5. Conclusions

We have identified *SLC2A1*, *TLE1*, *FAM83A*, *HMGA2*, *FBXO44*, and *MTRNR2L12* as biomarkers contributing to resistance in LUAD patients receiving AMG510 treatment. Additionally, we observed a correlation between AMG510 resistance and immunosuppression markers such as PDL1, reinforcing the significance of the immune microenvironment in tumor therapy. However, our study did not delve into the mechanistic aspects, such as elucidating the interactions between key molecules and the immune microenvironment that influence resistance. Exploring these mechanisms will be a crucial focus for future investigations. In summary, our findings offer promising targets for further research and drug development (e.g., antagonists and activators) in the treatment of LUAD.

## Data Availability

Datasets employed and/or scrutinized during this study can be procured from the respective author. The outcome data from the research can be accessed on websites such as TCGA and GEO.

## Supplementary Materials

The following supporting information can be downloaded at: https://www.mdpi.com/article/10.3390/ijms25031555/s1.
