# Supplementary material for: Bioinformatics and Experimental Validation for Identifying Biomarkers Associated with AMG510 (Sotorasib) Resistance in KRASG12C-Mutated Lung Adenocarcinoma
Source: Int J Mol Sci. 2024 Jan 26;25(3):1555. doi: 10.3390/ijms25031555 (PMC10855101; doi:10.3390/ijms25031555)
Supplement: Supplementary file 1 [file ijms-25-01555-s001.zip › ijms-2777022-SI.pdf]

Bioinformatics and Experimental Validation for Identifying Biomarkers Associated with AMG510 (Sotorasib) Resistance in KRAS^{G12C}-Mutated Lung adenocarcinoma

Peng Lin[1,†], Wei Cheng[1,†], Xin Qi[1], Pinglu Zhang[2], Jianshe Xiong[2*], Jing Li[1*]

Address:

[1]Key Laboratory of Marine Drugs, Chinese Ministry of Education, School of Medicine and Pharmacy, Ocean University of China, Laboratory for Marine Drugs and Bioproducts of Qingdao National Laboratory for Marine Science and Technology, Qingdao 266003, P. R. China.

[2]Faculty of Information Science and Engineering，Ocean University of China, Qingdao 266003, P. R. China.

GO and KEGG Functional Annotation

The annotation and visualization processes were performed utilizing the Metascape database (www.metascape.org). Our analysis focused on assessing the enrichment of differentially expressed genes in the Kyoto Encyclopedia of Genes and Genomes (KEGG) pathways and Gene Ontology (GO) terms. To establish statistical significance, we set criteria that included at least three overlaps and a P-value at or below 0.01.

Table S1. Primer sequences for RT-qPCR

SLC2A1	F:5′-GAGCAGCTACCCTGGATGTC-3′
	R:5′-GGCCACAAAGCCAAAGATGG-3′
TLE1	F:5′-GGCTGCACTCTCATAGTGGG-3′
	R:5′-AAGCAGACCTTGGAATCGGG-3′
FAM83A	F:5′-GTGGAGCTGTTTGACGAGGA-3′
	R:5′-CATCACAGGCTTGGAGGAGG-3′
HMGA2	F:5′-CCACTTCAGCCCAGGGACAA-3
	R:5′-TCCTCTCTTCTGAGGCGCTG-3′
FBX044	F:5′-ATCCTGCTGGAGCTGTTCAC-3′
	R:5′-GTTTCCAGAGGGTCACGAGG-3′

Table S2. Differentially expressed genes (up- and down-regulated)

**DEGs**	**Gene name**
**Upregulated (431)**	CD24, SLC16A7, BPIFB1, LGALS9, MAP2, TMEM176B, SLC44A4, TMC5, PROM1, ITM2A, CTSS, FAM3D, SLPI, LITAF, CLDN3, ALDH1A1, TTR, CREB3L1, TOX3, SORBS2, CDS1, FER1L6, PDZK1IP1, PLA2G10, ELAPOR1, GSTA1, CEACAM5, RETREG1, DHRS3, F2RL2, GALNTL6, MUC16, LINC00942, NPNT, GMDS, VTCN1, TC2N, MACC1, PLCL2, SCGB2A1, KRTCAP3, PSMB9, HLA-B, OCLN, SPINK1, ASRGL1, KIAA0319, ST6GAL1, VDR, DSTN, C2orf72, GDF15, CDR2L, PLAAT4, ANG, ARFGEF3, ZNF703, WFDC2, ELF3, CYP4F3, HRH2, AGT, TP53I11, CYB5A, SHROOM1, LINC02532, AP1M2, ONECUT2, TMED6, C5, SEMA5A, FOSL2, NCOA7, KCNS3, TRIM16L, SMIM31, THSD7A, MUC4, GALNT7, RASSF5, APOD, LYPD6B, VWA5A, MARCHF3, ITGB6, XBP1, MAST4, PTGR1, PHEX, CLDN7, MIR9-1HG, RCAN2, PGGHG, MECOM, CCL28, CFB, AL121839.2, CLDN1, ABCA12, LINC02381, CXCL16, NQO1, F5, MITF, CFH, NFKBIZ, ARRB2, MATN2, FAM102A, RNASE4, GOLPH3L, ARHGDIB, ECI2, CNR1, PLA1A, CHCHD7, MLXIP, EMBP1, ATP2C2, ADGRF1, CFI, BEX4, PIP, NCF2, TSPAN13, GSTP1, B2M, CXCL1, SEMA6A, FUT2, CCDC198, MTUS1, ADGRV1, C4orf19, AC131206.1, BAAT, CTDSP2,

	NUCB2, RHBDL2, OPHN1, ERMP1, FTH1, RNF217-AS1, EDN1, SLC29A1, SLC35C1, BMP2, CCDC18-AS1, UCHL1, SCRN1, SLC1A1, HOXA-AS2, GADD45G, CPLX2, CHP1, CLIC6, DCPS, HLA-E, GOLM1, CBR1, LCP1, AC006230.1, ACSS1, MPLKIP, BMP3, GLTPD2, ARHGEF17, DRAIC, AQP1, CPS1, REPIN1, COL4A4, SLC38A10, RND3, FXYD3, ADH1C, PLCXD2, RABGAP1L, GALM, MX1, GALNT3, ALOX5, IFNAR2, YIPF2, DMKN, NSF, SUSD6, LCN2, CRABP2, TPP2, BACE2, KIF26B, EMP2, KRT7, SORT1, MGAT1, SCN2A, HLA-A, TM7SF2, ARHGAP23, HS3ST1, FOXA1, NFIA, SPSB2, ABHD17A, CYP2B7P, AHSA2P, FAM133A, AC009407.1, KRT4, AKR1C4, ANKFN1, TRIP6, CBX6, SCAMP2, IRF2BP2, MMP7, TTC39A, IGFBP4, C1S, FTL, ZNF572, ANKRD36C, IFI27, RNF103, CACNA1H, MBOAT1, SLC22A17, PDE1B, FAM214A, VPS45, HES1, CYP4F11, COL4A3, C2orf15, SRGAP3, ABCA5, RRNAD1, TMEM125, BICDL2, EPB41L5, SLC25A29, QPRT, TMEM106C, ABHD11, GCLM, H2BC21, PAK1, PIGR, EPB41L1, PLCB2, DAPK1, EPB41L4A, CBR3, PARM1, LIMK2, SH3BGRL2, CCDC170, FOXP2, RAB40B, SMOC1, AL033397.1, C1orf216, ALDH3B1, AC019322.4, MGAT4A, HOXC6, TMEM9B, PHLDB2, GABARAPL1, UNC93B1, RPRML, CEBPA, VSTM2L, ZNF12, GLIS3, TLE1, PTPRF, AKR1C1, AR, PIK3C2B, CLDN4, BLCAP, CAT, ZER1, HLA-F, FILIP1L, AKR1C3, POU4F1, AC135050.6, KCNK5, GATM, NMRAL2P, ARMH4, PROC, IRF1, TALDO1, TNS3, TCEA3, SEPHS2, TMEM59, SYTL2, FRK, SLC40A1, SMIM4, PRR15L, IRF6, COL27A1, EFNA1, NRARP, SECTM1, AC010168.2, RAB11FIP5, AC004951.1, AC004870.2, ST14, PSMD11, CAMK1D, SAMD9L, PCED1B, TNRC6C, TRAPPC4, AL591895.1, ZNF277, ABL1, MBNL3, ADAR, TM4SF5, MYLIP, CLTRN, PLCB1, UBE2L6, CCDC7, RBM47, PDE5A, SLC48A1, AOC1, CD38, CD63, PCAT7, TMC4, LINC00632, MAP1A, RTN3, JUP, AC027117.1, PSMB8, TMEM230, TSPAN7, PSMB8-AS1, PARD3B, CCPG1, OSGIN1, SGSM1, IFIT3, CCNJL, AQP3, FSTL1, PRKAB1, LRIG1, DHRS7B, CDH1, SELL, LINC00342, PRRG4, PRKCB, SLC1A5, LASP1, HMGB3, NEURL1B, BLOC1S4, PRSS16, TACSTD2, RARRES2, GALNT6, NAA20, NCALD, SDCBP2, NORAD, PEG10, SCNN1A, SLC15A2, RNF113A, BANK1, TOX, ACOT13, LYZ, RUSF1, HNRNPH2, PRSS8, DPP3, PKDCC, SMAGP, GDA, MAL2, GUCD1, GULP1, FUT6, MBNL1, AC124319.1, ARSL, BCAS4, HYI, MGST1, HPN, FOLR1, HMGCS2, SSPN, LINC02503, ACADVL, TMEM106A,

	STARD10, EXPH5, PFKFB3, MAFG, DAB2, PIEZO1, SDCBP, PIM3, WNK4, ARPC3, C12orf75, MUC1, BLOC1S2, PDE3A, HNRNPA0, ESRRA, NCMAP, SNAPIN, ZNF853, PPP3R1
**Downregulated (403)**	CPE, CACNA2D4, LY6D, LAMA4, LRCH1, CST1, CYSRT1, CST4, S100A1, PDLIM4, PDLIM7, KCNJ12, S100A13, AC138230.1, UTRN, SLC20A1, CSGALNACT1, CHST6, HTRA1, TRNP1, ITGB3, CYP27C1, JCAD, GNG4, ITGA5, NET1, EVPL, MRPS6, GPER1, AC010198.2, GRK6, ITGA6, SLC5A3, ADGRG6, SPRED3, AC018629.1, LFNG, LTBP2, HRAS, ITGB4, HDAC11, TYRO3, FABP5, TESC, PACSIN3, PDE4B, CDCP1, ZBTB1, PPTC7, S100A16, UNC13A, AL161431.1, RGS10, SATB2, ATOX1, BMERB1, AP1S1, CDC25B, NT5E, DUSP5-DT, RAB31, NT5DC3, MASTL, FMNL1, SCG2, IGFBP1, GAS2L1, LGALS1, PRSS3, RNU5F-1, COL6A2, ACOT7, SUFU, ETV4, AXL, CCNG1, PNPLA3, BASP1, MOB3A, ARL14, DCLK1, PPP5C, NOS1, CAV1, SLC44A3-AS1, UBE2E2, NDE1, GPAT3, CAPG, TIAM1, SMOX, CPVL, CD320, CEP19, COL6A1, POLRMT, SPRYD7, SLC30A1, ADIRF, ROBO1, PCID2, SULF2, OSBPL11, ADAM8, ITPRID2, SH2D2A, AC010624.5, IL27RA, HSD17B1, ZFYVE19, SEC14L2, GRAMD1B, MORC4, TNNT1, NPW, GAREM1, CDYL2, EFNB2, MT-TT, ONECUT3, ZNF524, DDB2, MMP28, LINC02595, CDKN2AIP, EMP3, KANK2, GAL3ST1, PLOD3, PLEKHG5, CAPRIN2, ERRFI1, A4GALT, S100A2, HPGD, ASIC1, COL17A1, FAM210A, TOB1, VCAN, STMN2, FBN1, CADM1, DLGAP4, CSGALNACT2, NOSTRIN, KCNC4, ID2, PCCA-DT, RUNX3, TCF7L2, STC1, SOD3, MXRA7, PRR7, BCL9L, LRRC8E, RORA, LINC01239, RRAS, PPP1R3C, BCL2L1, ADAM12, PIP5K1C, TCN1, NUP62CL, ZNF581, DRG1, TGFB1, SSTR5-AS1, FJX1, KIAA1549, SLC35F2, AP2S1, PLAT, EHD2, ANKRD18B, LRRC28, MCM8, AL035252.5, SDR16C5, ABO, BIRC7, TBC1D4, CFAP251, AHNAK2, DUSP6, SLBP, INTS2, CFD, TPK1, FBXO44, CTNNAL1, SOCS6, PALM2AKAP2, NNMT, CSKMT, BAIAP2L2, RAB27B, HMGA2, PAQR5, TNFRSF18, HBEGF, TMCO3, MT-TS2, JAG1, TNFRSF10D, JOSD2, FOXC2, C10orf88, YBEY, MDFI, SEMA3A, SERPINB8, FAM89B, XRCC3, NUDT18, AFAP1, NAB1, ZNF219, PLEKHG2, AHR, ADAM19, LIAS, LYNX1, TOP1MT, PAEP, CDH19, STXBP1, CCDC102A, RRP9, AL035530.2, NES, HSD17B2, TMEM209, CARD10, TCEA2, FUT11, NOSIP, TRIM7, RPL29, EREG, MAPKBP1, PPAN, BICDL1, ZNRF3, CHEK1, ITCH, ELK4, GCAT, RAP1GAP2, CEP131, SENP3, EHBP1L1, FAM83A,

	ENPP1, TNFSF15, PPIB, ZRANB3, GTPBP10, FAM20C, PHF10, TM4SF18, MIER2, MT-TW, PRICKLE3, TMEM158, CASC19, MT-TV, MT-TH, DUS2, SLC2A13, KIAA1841, AL035252.3, ZNF114, B3GNTL1, HAGHL, ABHD17C, SOGA1, CCDC124, CIBAR1, MT-ATP8, NUDT8, DEAF1, RARA, SHLD1, PCCA, TFAP2C, ARHGAP29, OAS1, CDKN2AIPNL, MTRNR2L11, RAET1E, ASNSD1, WDR75, SNCG, PANX1, AREG, FMN2P1, MBLAC2, MTRNR2L7, PTPA, FXYD2, PES1, FAH, MTRNR2L12, STARD8, C22orf46, BOK, IFT88, PIGZ, DGCR5, ZFYVE9, DOCK6, NUDT17, RAD9A, PHLDA1, RBPMS2, GPR153, ITPRIP, KDM3A, TXNRD2, CCDC9B, DOCK4, TTC28-AS1, AC026356.2, NFKBIL1, LAMC2, UIMC1, MAD1L1, PTDSS2, SFT2D3, ASB7, FUOM, TUBB6, PRSS23, MIR222HG, FAT1, PPP1R1B, CLIC4, INO80B, FARP2, LY6E, CDA, FHIT, ANKRD37, RIN1, MT-TY, CALB2, B3GNT6, MRC2, SLC25A24, SPTLC3, GPR137, C16orf74, ITPR2, MTCL1, PCSK6, BLOC1S5, IL17RA, TNNI3, CUTC, SAMD1, BCAR3, MYBL1, APOC1, SHH, SERPINA1, SNORD13, AC002070.1, MTND4P12, CRAT, SYT12, SPINT1, LAMA3, INPP5F, FKBP8, TRABD2A, ESPN, AVEN, MROH6, AC010735.2, SMTN, C19orf25, MUC5B, ELFN2, NUDT14, CLIP2, ACSL5, MUC2, CYP24A1, CPNE8, ABCA2, TNS4, SLC2A1, MT-TE, FBXO2, CD55, AC092828.1, MITD1, VPS18, PAQR7, EEF1A2

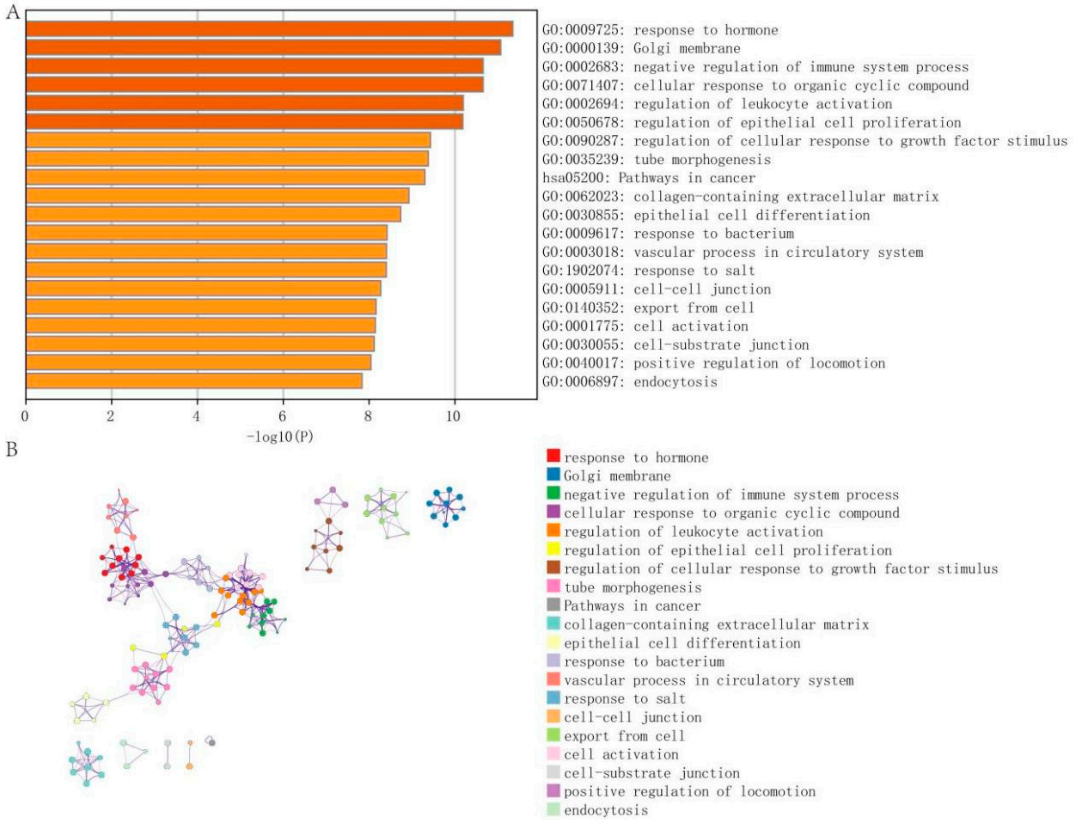

Figure S1. Functional enrichment of DEGs by metascape enrichment analysis.

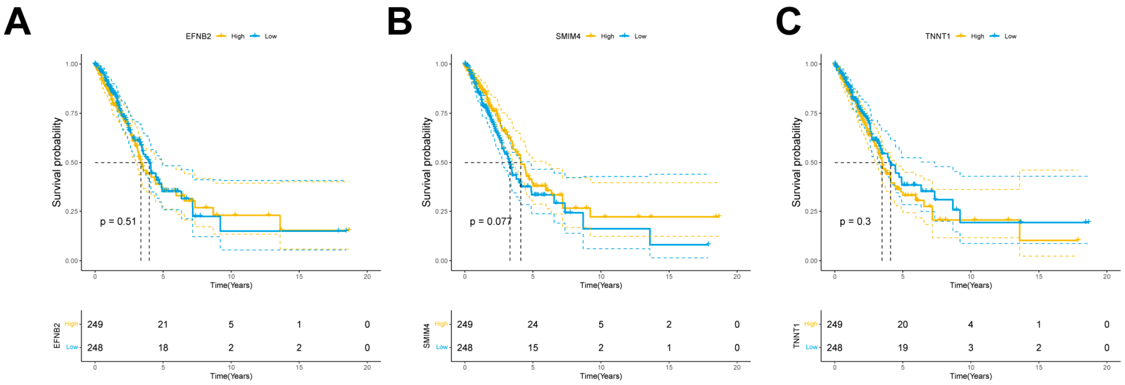

Figure S2. The expression levels of EFNB2, SMIM4, and TNNT1 and their association with LUAD patient prognosis.

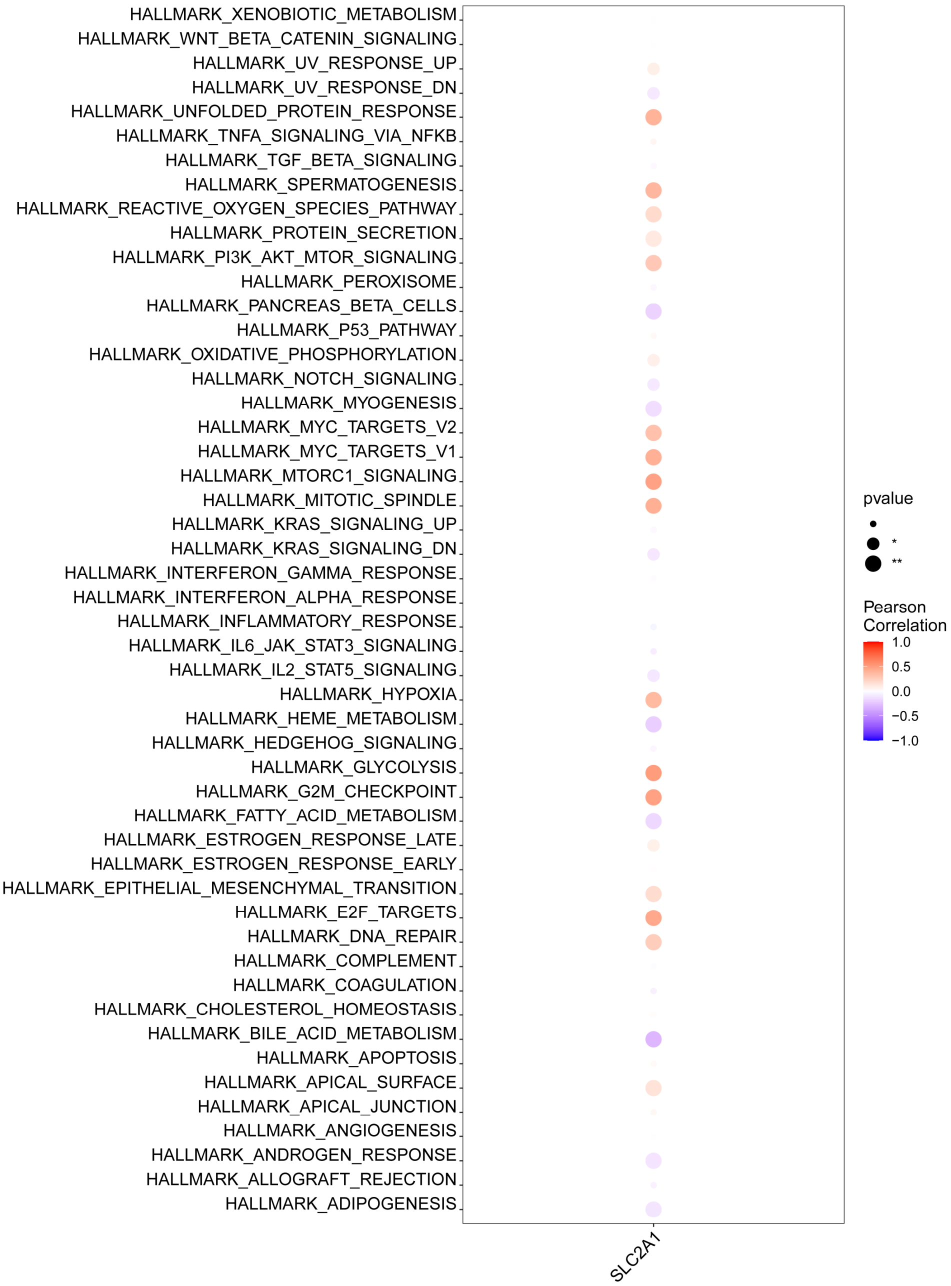

Figure S3. SLC2A1 correlates with LUAD-related signalling molecules

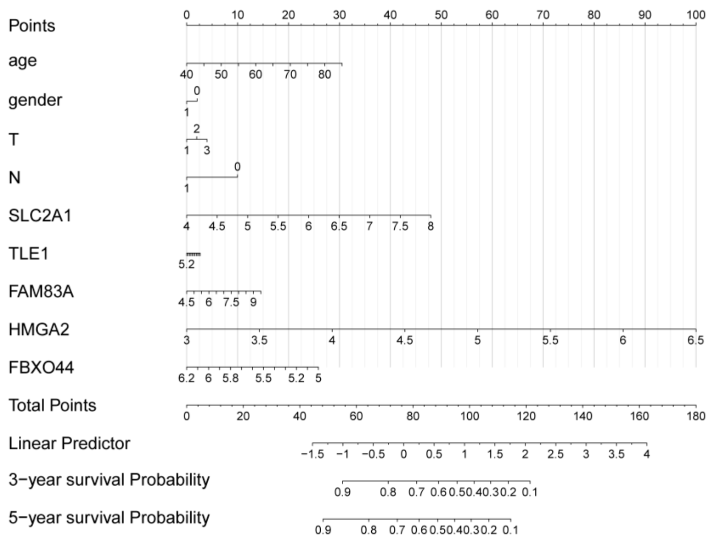

Figure S4. Nomogram and calibration curves for prediction of prognosis of LUAD patients (GSE30219)

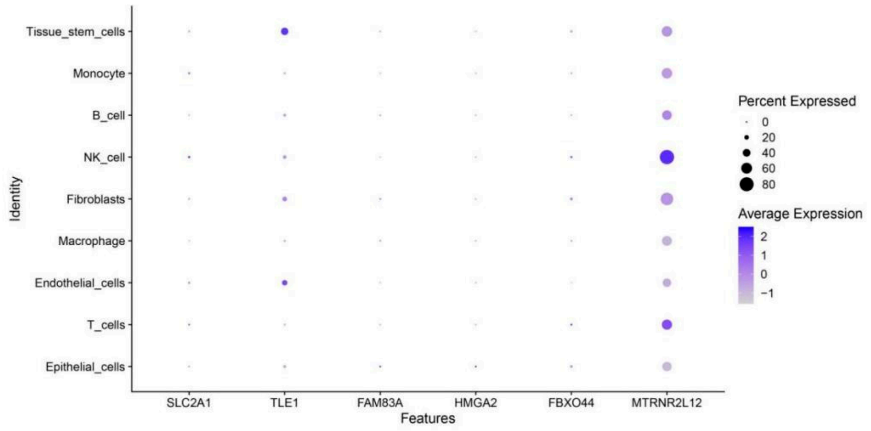

Figure S5. Distribution of key genes for drug resistance in 9 different cell types.
